# Understanding user intention to use online study rooms: an integrated model of technology acceptance and flow experience

**DOI:** 10.3389/fpsyg.2026.1935259

**Published:** 2026-10-09

**Authors:** Shangjie Yuan, Jun Wang

**Affiliations:** Hunan Institute of Science and Technology, Yueyang, China

**Keywords:** online study room, technology acceptance model (TAM), Flow Theory, intention to use, user behavior

## Abstract

**Introduction:**

Online study rooms have gained popularity among users for their flexibility and interactivity. However, there remains a lack of in-depth analysis of factors that influence user behaviors in these virtual learning environments. This research delves into the determinants of user behaviors in the context of online study rooms by integrating the technology acceptance model (TAM) and the flow theory.

**Methods:**

Structural equation modeling (SEM) with PROCESS moderation analysis was performed to analyze 389 valid responses.

**Results:**

The research findings manifest that: (a) external variables (i.e., learning convenience, interactivity, and the incentive and constraint mechanism) significantly influence users’ perceived ease of use and perceived usefulness of such platforms; (b) perceived ease of use has a direct and positive influence on the intention to use (supporting H2), while perceived usefulness has no direct influence (rejecting H3); (c) flow experience is a key determinant of intention to use; and (d) gender and habituation significantly moderate the relationships between perceived ease of use, perceived usefulness, flow experience, and intention to use.

**Discussion:**

By demonstrating that emotional immersion (flow) complements rational utility evaluation in using technology, this research extends the boundary conditions of TAM and offers a dual-pathway framework for understanding user behaviors in digital learning environments. Our research results offer data support for optimizing the design of online study rooms, theoretical evidence for understanding user behaviors in novel digital learning environments, and practical implications for developers of such platforms and personnel working in this domain.

## 1 Introduction

With the increasing prevalence of the Internet and the growing demand for flexible learning, online study rooms, a novel digital learning environment, are mushrooming quickly. This innovative learning environment highlights the flexibility and efficiency of independent learning, where users can concentrate on learning tasks on online platforms, such as Zoom or YouTube, and users can engage in discussions and share insights with other users regarding their learning progress (Wang et al., 2024).

Since the emergence of online live streaming “study with me” on YouTube in 2017, online study rooms have gained popularity quickly on Chinese platforms such as BiliBili and Douyin. Statistics revealed by BiliBili display that in 2018, the number of participants in online learning live streams surged to 18.27 million, the cumulative live streams started hit 1.03 million, and the total hours dedicated to such live streams exceeded 1.46 million hours, positioning this type of live streaming as the predominant genre in terms of streaming hours. Since July 2021, the number of participants in “Learning Companions” live streaming has exceeded 327 million, manifesting the massive potential of online study rooms.

The intricate relationship between education and information technology has made online learning an integral part of education reform. Especially during the COVID-19 pandemic, online learning emerged as the principal pedagogical method adopted by numerous educational institutions, which greatly facilitated the alteration of learning methods. Backed by live streaming technologies, online study rooms refer to virtual learning environments accessible via devices such as smartphones and computers, where users can conduct real-time interaction with peers across diverse geographical locations who share a synchronous viewing experience (Zhang et al., 2024). This learning mode not only transcends the temporal and spatial limits of traditional learning but also enhances social presence and interactivity, and cultivates a collective learning atmosphere (Lim et al., 2023).

Despite the rapid growth of research on online study rooms in China, particularly in the wake of the COVID-19 pandemic, the predominant methodologies employed in existing studies have been qualitatively oriented, such as participatory observation and in-depth interviews, and there is a lack of quantitative studies. The existing literature on online study rooms can be categorized into three types: first, studies focusing on the operational dynamics and user demographics of online study rooms (Xu et al., 2021). Second, investigations on user motivations and the intrinsic factors that encourage participation in such online study rooms (Lim et al., 2023), third, studies evaluating the effects of online study rooms on learning outcomes and their efficacy (M. Kim, 2022). Nevertheless, a research gap persists regarding factors that influence the behavioral intention to use online study rooms, especially in the aspects of technology integration and emotional drivers (Venkatesh, 2022).

Technology Acceptance Model (TAM) serves as a classical theory for understanding user behavior in accepting technologies. Its key variables, perceived usefulness and perceived ease of use, have been widely applied to elucidate users’behavioral intentions (Davis, 1989). In recent years, TAM has been extensively employed in the context of digital learning (Lisana, 2023; Afacan Adanır and Muhametjanova, 2021) However, TAM pays little attention to emotionally driven factors, which makes it inadequate to explain users’ continuous participation in online learning environments (Venkatesh, 2022)，and fails to investigate the moderating effects of individual differences such as gender and habituation. In recent years, the Flow Theory has garnered attention in studying online learning environments, which unveils the significant influence of emotional factors on user behavior (Hsu et al., 2012).

By integrating TAM and the Flow Theory into the theoretical framework, this research aims to systematically elucidate users’ behavioral intention to use online study rooms. Unlike traditional TAM studies that primarily focus on rational utility evaluation, our integrated framework posits that emotional engagement, captured through flow experience, serves as a complementary mechanism that works alongside, and in some contexts supersedes, cognitive assessments of usefulness and ease of use. Specifically, this research not only seeks to explore the roles of perceived usefulness and perceived ease of use as rational driving forces of user behaviors but also introduces flow experience as a mediating variable that highlights the critical role of emotional factors in making decisions. Furthermore, this research examines the moderating effects of gender and habituation on user behavior, which addresses the existing gap in the literature concerning emotionally driven factors and the moderating effects of individual differences. Structural equation modeling (SEM) was employed to analyze 389 valid responses. By demonstrating that emotional engagement complements rational technology evaluation in shaping decisions, this research not only enriches the application scenario of TAM but also offers practical insights for the design and optimization of online study rooms. The research results can help platform developers better understand user demands and design features to improve user engagement and satisfaction (Lim et al., 2023).

The structure of this study is organized as follows: the first part is the introduction; the second section constitutes a literature review that examines the current status of research on online study rooms and TAM; the third part outlines the research framework and hypotheses; the fourth part introduces the research methods, including questionnaire design and data collection processes; the fifth part presents the research results; the sixth part discusses research findings and implications; the last part centers around the conclusion, limitations, and future research directions.

## 2 Literature review

### 2.1 Online study rooms

As a novel learning environment, online study rooms have garnered much attention in recent years. Established by digital technologies, these spaces support coordinated, independent, and immersive learning experiences (Sage et al., 2021). From the perspectives of Situated Learning Theory and Distributed Cognition Theory, online study rooms offer learners cognitive opportunities for embodied cognition by simulating authentic learning scenarios, which can enhance their contextual understanding of knowledge (Chou and Liu, 2005).

Take China’s BiliBili online study rooms as an example. This platform leverages live streaming technologies to enable individual streamers or groups of users to create a virtual learning environment using their electronic devices, such as smartphones or computers. Learners can showcase diverse learning activities, ranging from note-taking and problem-solving to recitation and typing, via the real-time “lives streaming” feature. The audience can participate by watching these activities in real time, fostering a collective learning experience where participants can engage with content simultaneously despite geographical dispersion. Features of bullet comments and virtual avatars enable online study rooms to enhance social presence and simulate a co-present experience, which further boosts interaction and exchange among learners. To cultivate a good learning atmosphere, the streamer usually pinpoints clear learning objectives, timelines, and motivational slogans. Users can send bullet comments to document their engagement, encourage each other, and share insights to foster an environment where supervision, self-motivation, and companionship are available.

Previous studies in this field have mainly focused on the foundational aspects of online study rooms, such as platform stability and user interface design. As research deepens, scholars shift toward examining the relationship between such platforms and user demands, and their influence on learning outcomes (Liu et al., 2022) pointed out that effective emotional support in online teaching environments can offer empathy, understanding, motivation, and encouragement to learners. While prior research has offered valuable insights, most of them are qualitative studies that lack accurate quantitative analysis. Moreover, existing literature fails to delve into how online study rooms, a novel learning medium, trigger and influence intention to use, and the endeavors to investigate the positive influences of emotional factors on intention to use remain insufficient, leading to gaps in explaining user behaviors within this context (Fathali and Okada, 2018).

### 2.2 Theoretical frameworks

#### 2.2.1 Tam

First proposed by Davis (1989), TAM is an essential theoretical framework for examining user acceptance behaviors toward information technologies. Its application spans multiple fields, including information systems, informatics, and library science. Perceived usefulness and perceived ease of use are two primary variables in TAM used to explain technology acceptance behaviors Davis (1989). Posited that perceived usefulness refers to users’ cognition of whether a given technology is useful, and perceived ease of use pertains to the perception regarding the simplicity of using a particular information system. Subsequent studies have sought to refine this model by including the influences of external variables on perceived usefulness and perceived ease of use. Notably, (Venkatesh et al., 2003) extended the TAM2 framework by including factors that influence perceived ease of use.

In the context of online learning environments, the effectiveness of TAM has been extensively validated. Motaghian et al. (2013) employed a comprehensive model integrating information systems, and psychological and behavioral factors to evaluate teachers’ intention to adopt a learning system. Their research results show that perceived usefulness and perceived ease of use notably improved teachers’ behavioral intention to use online study systems, and PU was found to be the most influential factor. Arteaga Sánchez et al. (2014) investigated students’ intention to use Facebook to aid learning, and the results showed that perceived usefulness, perceived ease of use, and facilitating conditions have significant positive influences on the adoption of Facebook. To this end, TAM is deemed an effective framework for investigating the adoption and behavioral intention to use online study rooms.

#### 2.2.2 Flow theory

The Flow Theory was initially conceptualized by Csikszentmihalyi (2014) which delineates a psychological state when an individual is completely immersed in enjoyable activities and temporarily ignores other stimuli in the surroundings. This state of intense concentration is marked by several key features: the individual’s complete concentration, a distorted perception of time, and the intrinsic satisfaction derived from the activity. As Internet technologies advance, the Flow Theory is widely applied in a host of areas, such as social media usage behaviors (Hyun et al., 2022), information technology (Kim et al., 2019), and online gaming (Erhel and Jamet, 2019), It is particularly valuable in explaining individual behavioral motivations and flow experiences.

Additionally, the Flow Theory has been applied in learning contexts that require a higher degree of individualism. Hoffman and Novak (1996), first introduced the Flow Theory into online environments, and they asserted that flow could boost increased learning, perceived behavioral control, exploratory mindset, and positive subjective experiences. Hoffman and Novak (2009) described the flow state as a cognitive state and elucidated the seamless response, interactivity, intrinsic enjoyment, the loss of self-consciousness, and self-reinforcement in online environments. Research by Cheng and Jiang (2020) regarding reading and learning behaviors illuminated that the learning efficiency and outcomes in an immersive environment outperform that of a normal environment.

#### 2.2.3 Integrating TAM and Flow Theory: a complementary framework

The integration of TAM and Flow Theory in this study stems from the recognition that technology acceptance of users in immersive learning environments involves both cognitive evaluation and emotional engagement. While TAM explains it through rational assessment of instrumental benefits (perceived usefulness) and operational ease (perceived ease of use), Flow Theory captures the experiential and affective dimensions of technology use. Specifically, the state of deep concentration, intrinsic enjoyment, and temporal distortion that characterizes optimal learning experiences (Csikszentmihalyi, 2014; Hoffman and Novak, 2009).

Flow Theory explains beyond traditional TAM models for three primary reasons. First, online study rooms are not merely functional tools, but immersive environments designed to simulate physical learning spaces and foster social presence. Users’ engagement extends beyond instrumental utility to include experiential rewards such as a sense of companionship, collective motivation, and emotional immersion. Second, sustained learning behaviors, particularly in self-oriented contexts, require not only perceived usefulness but also intrinsic motivation and enjoyment that are central to flow experiences (Agarwal and Karahanna, 2000; Ryan and Deci, 2000). Third, the social and interactive features of online study rooms are specifically designed to induce flow states, suggesting that flow may serve as a mechanism through which platform features translate into behavioral intention.

The relationship between cognitive evaluation of TAM and emotional immersion of Flow Theory can be understood through a dual-process framework. Perceived usefulness and perceived ease of use represent the hard cognitive pathway. Users rationally assess whether the platform helps them achieve learning goals. Flow experience represents the soft affective pathway. Users emotionally engage with the platform, experiencing deep concentration and enjoyment. Importantly, these pathways are not independent. External platform features can simultaneously influence both cognitive evaluations and emotional experiences to synergistically effect on behavioral intention.

### 2.3 Research hypotheses

#### 2.3.1 Perceived usefulness and perceived ease of use

Based on the Theory of Reasoned Action (TRA) proposed by Fishbein and Ajzen, Davis (1989) developed TAM. Currently, TAM has been recognized as the best framework to understand acceptance behaviors associated with information technologies (Venkatesh, 2022). (Wang et al., 2020). Perceived usefulness and perceived ease of use are two critical constructs for elucidating users’ behavioral intentions. Specifically speaking, perceived usefulness refers to the degree to which users believe that using an application can improve their work or learning outcomes. Perceived ease of use refers to the degree to which users believe that using an application or equipment requires few efforts (Alturki and Aldraiweesh, 2023). For example, in the learning context of the metaverse, perceived ease of use is deemed a crucial determinant of technology acceptance (Al-Adwan et al., 2023).

In this research, perceived ease of use is defined as the degree to which users of online study rooms do not encounter complex technological operations or excessive cognitive burdens. To be more specific, a higher level of perceived ease of use correlates positively with the behavioral intention to use online study rooms. Previous research on TAM has substantiated that perceived ease of use serves as a prerequisite for perceived usefulness (Nguyen et al., 2024; Türker et al., 2022). In this study, perceived ease of use can enhance users’ belief of users’ belief in the potential benefits of online study rooms, such as improved learning efficiency and heightened concentration. Given the above analysis, we propose the following hypotheses:

*H1*: Perceived ease of use of online study rooms positively influences perceived usefulness.

*H2*: Perceived ease of use of online study rooms positively influences their intention to use such platforms.

In the current research, perceived usefulness is defined as users’ perception of learning benefits derived from using online study rooms, which is mainly presented in two aspects. First, the improvement of learning efficiency. With real-time discussion and sharing of learning progress, online study rooms allow users to access information and resolve inquiries more efficiently. For example, users can interact with streamers via bullet comments to address questions occurring in the learning process in real time, thus improving their learning outcomes (Zhang et al., 2023). Second, the cultivation of learning habits. Through the check-in feature, objective setting, and the incentive and constraint mechanism, users can better establish and maintain beneficial learning habits, which not only enhances users’ motivations to study but also improves their intention to use by fostering a sense of achievement gained from goal fulfillment. Moreover, higher perceived usefulness is likely to be associated with a greater intention to use online study rooms, as users recognize the tangible benefits of improved concentration, optimized learning strategies, and enhanced learning outcomes (Kim, 2022). To this end, this research hypothesizes that:

*H3*: Perceived usefulness of online study rooms positively influences their intention to use such platforms.

#### 2.3.2 Learning convenience

Learning convenience is defined as students’ capacity to engage in learning activities without temporal and spatial constraints (Lisana, 2023). Pramana (2018) indicated that the inconvenience of attending physical classrooms is a key factor affecting university students’ intention to use Mobile Learning platforms. Several researchers have also highlighted the significance of convenience in fostering students’ willingness to adopt Mobile Learning in higher education institutions (Qashou, 2021; Saroia and Gao, 2019).

In this research, learning convenience is defined as the ease of access to online study rooms, which are no longer hindered by the temporal and spatial limits of conventional learning environments (Lisana, 2023). Compatible with various devices, online study rooms allow users to switch seamlessly between mobile phones and computers, which alleviates the cognitive burden of adapting to different technological interfaces. Users can customize their learning content and pace to meet their individual needs, which enhances the flexibility and autonomy of learning (Pramana, 2018). Streamlined operational procedures contribute to a perception of convenience and further enhance users’ perceived ease of use. For example, the multi-device compatibility of online study rooms can mitigate the cognitive burden of adapting to different technological interfaces. The flexible learning time options, such as 24-h live streaming, enable users to engage in learning activities without changing their schedules, which can lower the psychological barrier to using such technology (Saroia and Gao, 2019). When autonomy is enabled, for example, when users can choose their learning content, they will perceive less external control and pressure and master the technology with greater ease (Ryan and Deci, 2000). Based on the above analysis, we propose the following hypothesis:

*H4*: Learning convenience positively influences perceived ease of use.

The learning convenience offered by online study rooms improves users’ recognition of the system’s practicability through the optimized allocation of learning resources. Without spatial constraints and the necessity of commuting, online study rooms save users’ time and energy, and enable users to dedicate more time to achieving their learning objectives, which can lead to better learning outcomes, such as increased concentration duration. Hussein and Hilmi (2021) highlighted the significance of convenience in online learning environments and pointed out that convenience greatly contributes to improving user engagement and satisfaction. Furthermore, the autonomy enabled by customizing learning plans that cater to user demands strengthens users’ perception of the alignment between system functions and individual objectives, which can enhance their evaluation of usefulness (Collis and Moonen, 2011). The empirical study conducted by Lee (2010) further corroborates that flexible learning procedures (such as the effective use of fragmented time), facilitated by convenience, can significantly improve users’ recognition of the effectiveness of online learning. Users deem the system as a practical tool for achieving their goals. According to Zhang et al. (2023), convenience significantly influences users’ perceived usefulness of online educational platforms. Given the above analysis, it is hypothesized that:

*H5*: Learning convenience positively influences perceived usefulness.

#### 2.3.3 Interactivity

Interactivity refers to the extent of two-way communication experienced by users when interacting with the system, content, or other users. Prior research has highlighted the critical impact of interaction quality and frequency on online learning satisfaction (She et al., 2021). Burgoon et al., (2000) posit that interactivity can be understood through the qualitative aspects of users’ experiences during interactions, such as their level of engagement, mutual participation, and personalization. Kamoyo et al. (2025) research shows that e-learning platforms with high interactivity can enhance users’ perceived ease of use and usefulness and foster a positive attitude among students.

In the context of online study rooms, interactivity manifests through bullet comments, live chats, and the sharing of learning progress. By minimizing operational barriers, interactivity can improve users’ perceived ease of use of such platforms. Specifically, features like bullet comments and real-time feedback can streamline the interactive process between users and the system, and the ability to engage in discussions with a single click and the auto-synchronization of learning progress diminish users’ cognitive burden of using such systems (Song and Zinkhan, 2008). Moreover, high-quality information exchange, supported by well-refined bullet commenting rules and prompt responses to inquiries, optimizes the user experience and simplifies the mastery of such systems (Roy Dholakia and Zhao, 2009). To this end, this research hypothesizes that:

*H6*: The interactivity of online study rooms positively influences perceived ease of use.

Lee (2010) demonstrated that human-to-human interaction significantly enhances users’ perceived usefulness of ACG (animation, comic, and game) social media sites. In this study, interactivity is posited to improve perceived usefulness by enhancing learning support functions. For example, real-time discussion and coordinated problem-solving efforts enable users to quickly access learning resources and tackle complicated problems, which directly improves learning efficiency. Meanwhile, personalized interactions, such as streamers’ content adjustments in live streaming based on user demands, can reinforce users’ recognition of the system’s value and their belief that online study rooms are effective tools for achieving their learning objectives. Therefore, this research proposes the following hypothesis:

*H7*: The interactivity of online study rooms positively influences perceived usefulness.

In examining the relationship between interactivity and immersion, scholars have posited that interactivity can effectively elicit consumers’ sense of immersion within digital environments (Klingenberg et al., 2024). This is attributed to the fact that engaging with interactive elements significantly enhances psychological engagement among consumers (Roy et al., 2023). Furthermore, Kowalczuk et al. (2021) have demonstrated that interactivity not only positively influences immersion but also contributes to user enjoyment of augmented reality (AR) and their intention to reuse it in e-commerce contexts. Based on the above analysis, this research proposes that high levels of interactivity can significantly influence users’ flow experience. Thus, it is hypothesized that:

*H8*: The interactivity of online study rooms positively influences the flow experience.

Future research may further distinguish between synchronous and asynchronous interactivity to examine their differential effects on flow experience and behavioral intention.

#### 2.3.4 Incentive and constraint mechanism

As a fundamental component of behavioral monitoring mechanisms, the incentive and constraint mechanism functions as an objective-oriented feedback system in educational psychology and information technology. Khaldi et al. (2023) identified that PBL elements (points, badges, and leaderboards), levels, and feedback are the most frequently adopted gamification elements in e-learning systems within higher education. These elements facilitate social comparisons among learners through leaderboards and drive them to ascertain their positions within peer groups. It is also a competitive mechanism can significantly improve learning engagement. Meanwhile, Kluger and DeNisi (1996) posit that instant feedback on learning progress and outcome evaluation can help learners dynamically adjust their strategies and enhance their self-efficacy.

In online learning scenarios, such as the BiliBili online study rooms, the incentive and constraint mechanism manifests through learning time leaderboards and instant feedback on concentration. Leaderboards can clearly display learning outcomes and assist users in setting specific objectives. The change in rankings indicates individual progress and can foster a sense of competitiveness. Moreover, dynamic data also help users to refine their learning strategies and improve their efficiency.

The functions of leaderboards and concentration supervision of online study rooms can streamline the interactive processes between users and systems. Rapid rank checks and instant feedback on learning progress can alleviate users’ psychological burden of using technologies. Besides, the instant feedback mechanism can optimize user experience by offering real-time evaluations of learning progress and outcomes. To this end, the system is suitable for analysis through the lenses of TAM and the Flow Theory. As the external variable, the incentive and constraint mechanism can influence users’ perceived ease of use and usefulness of the system and their flow experience, thereby indirectly promoting their intention to use such systems. To this end, this research hypothesizes that:

*H9*: The incentive and constraint mechanism positively influences users’ perceived ease of use of online study rooms.

Wiyono et al. (2021) discovered that the incentive and constraint mechanism has a significant influence on performance-based learning. In the scenario of online study rooms, this mechanism can improve perceived usefulness by providing robust learning support. For example, learning time leaderboards and concentration supervision enable users to quickly access learning resources and address their problems, thereby directly improving their learning efficiency. By delivering learning support and personalized services, users’ recognition of the system’s value will be significantly increased, which can further improve perceived usefulness. In essence, if the constraint mechanism successfully enhances learning motivation and performance, the goal of supervision is realized. To this end, this research hypothesizes that:

*H10*: The incentive and constraint mechanism positively influences users’ perceived usefulness of online study rooms.

According to the Flow Theory proposed by Csikszentmihalyi (2014), in this research, supervision and incentives create a sense of belonging and enjoyment by stimulating emotional engagement (such as bullet comment encouragement and virtual avatar coordination) and offering continuous external stimulus (such as learning progress leaderboard and bullet comment check-in). These elements promote prolonged concentration and enhance the flow state. Real-time interactions and objective-oriented feedback mechanisms can significantly improve the flow experience. Therefore, it is hypothesized that:

*H11*: The incentive and constraint mechanism positively influences users’ flow experience in online study rooms.

#### 2.3.5 Flow experience

The concept of flow experience was first proposed by Csikszentmihalyi (1975), which pertains to the mental state of concentration and a distorted sense of time perception when an individual is deeply engaged in an activity. In information technology, Hoffman and Novak (2009) introduced this concept to online environments and highlighted that flow experience can enhance users’ sense of control and exploratory behavior via technological features such as interactivity and remote display capabilities. Extant research has indicated that flow experience can significantly improve the intention to use (Ozkara et al., 2017), especially in the context of online learning, where flow experience can enhance the enjoyment of learning (Guo and Zhang, 2024), boost cognitive absorption and ultimately lead to behavioral intentions (Agarwal and Karahanna, 2000).

In this study, online study rooms leverage virtual simulation technologies to simulate physical learning environments, where users can enjoy a quiet learning atmosphere without interruption. Therefore, users’ concentration and learning motivation can be improved. Besides, the design of online study rooms can harness customizable settings and interactive functions (such as real-time discussions and the sharing of learning progress) to further improve users’ flow state. Given the above analysis, this research proposes the following hypothesis:

*H12*: The flow experience of online study rooms positively influences the usage intention.

(see Figure 1)

**Figure 1:**
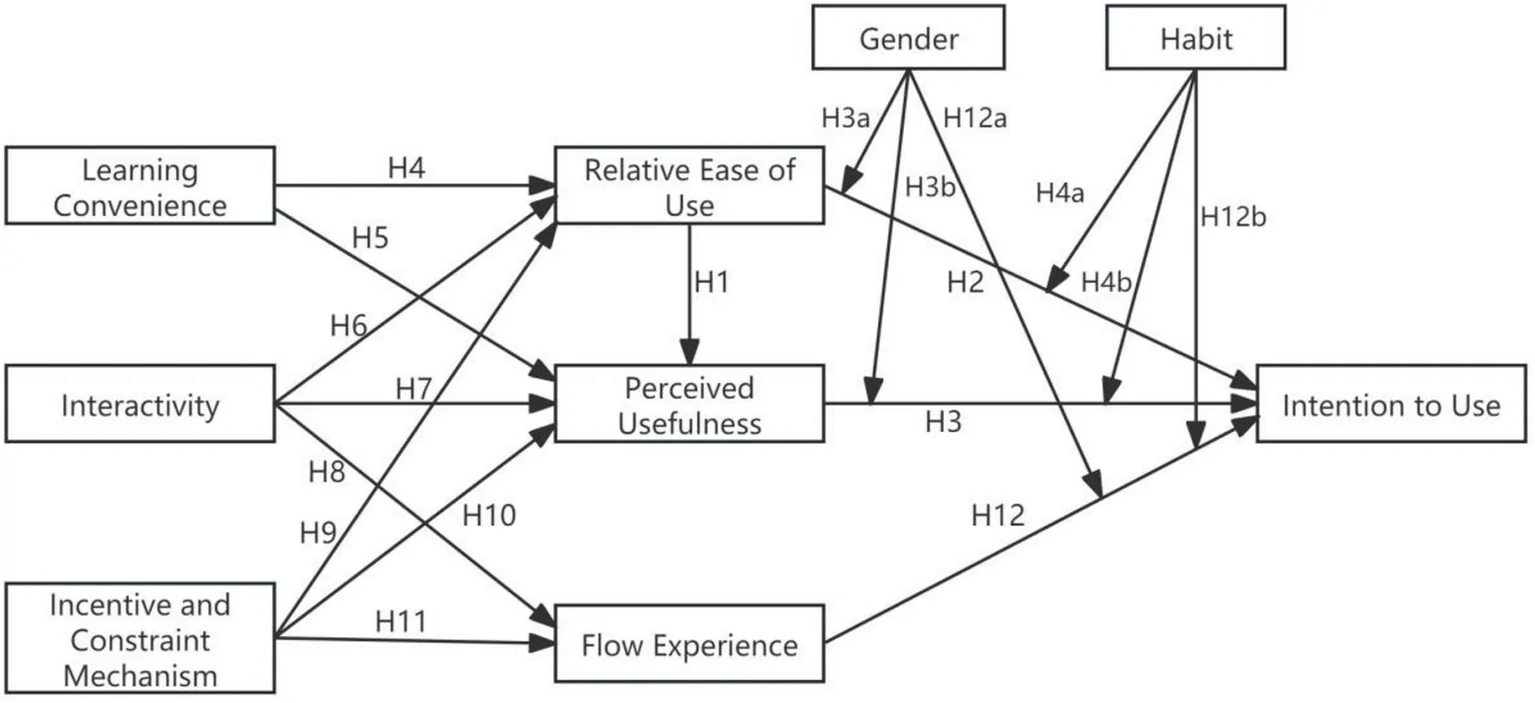
Theoretical framework.

#### 2.3.6 The moderating effects of gender and habits

Gender differences in this research refer to the different preferences and behavioral patterns exhibited by men and women when engaging with e-learning platforms. Drawing on Social Role Theory (Eagly and Wood, 1999), gender differences in technology acceptance can be understood through socially constructed role expectations that shape cognitive processing and decision-making strategies. Specifically, Female users tend to prioritize interactivity and user experience, while male users are inclined to emphasize practicability and functional design (Hoffman and Novak, 2009; Venkatesh et al., 2003). These gender differences could moderate the influence of perceived usefulness and perceived ease of use on the intention to use. Specifically speaking, female users may tend to enhance learning experiences through interactive features, such as bullet comments and real-time discussions, while male users may be more concerned about whether the platform can directly improve learning efficiency and outcomes. Consequently, it is posited that gender can moderate the influences of perceived ease of use and perceived usefulness on intention to use. Specifically, the positive influence of perceived ease of use on intention to use may be stronger for female users, whereas the positive influence of perceived usefulness on intention to use may be weaker for male users, as they tend to prioritize instrumental benefits that are less salient in emotionally-driven learning contexts.

Therefore, we propose:

*H3a*: Gender moderates the relationship between perceived ease of use and intention to use,such that the positive effect is stronger for female users.

*H3b*: Gender moderates the relationship between perceived usefulness and intention to use, such that the positive effect is weaker for male users.

Habituation refers to the automatic influence of users’ prior experiences on their current behaviors (Ryan and Deci, 2000). Drawing on Dual-Process Theory, habituation operates through System 1, an automatic, intuitive, and effortless cognitive processing that bypasses deliberate evaluation (Kahneman, 2011). When users develop habituation to certain learning routines, their decision-making processes shift from deliberate evaluation to automatic activation of established behavioral scripts.

In the context of online study rooms, habituation can influence user acceptance through two primary mechanisms. First, users who have been familiar with similar learning tools or established offline study routines may adapt to online study room functions more quickly, but their automatic behavioral patterns may reduce sensitivity to platform features. For perceived ease of use, highly habituated users may not evaluate ease of use consciously. Instead, they automatically engage with the platform based on established routines, which weakens the influence of ease perceptions on intention. Second, habituation can reduce cognitive elaboration, users who are highly habituated to certain learning contexts may rely on automatic decision-making rather than carefully evaluating platform usefulness, thereby weakening the relationship between perceived usefulness and intention to use.

*H4a*: Habituation negatively moderates the relationship between perceived ease of use and intention to use, that is, the positive effect is weaker for users with higher levels of habituation.

*H4b*: Habituation negatively moderates the relationship between perceived usefulness and intention to use, that is, the positive effect is weaker for users with higher levels of habituation.

Flow experience refers to the state of complete immersion and enjoyment of using a technology. Though flow experience itself exerts a direct influence on the intention to use, gender and habituation can also moderate the relationship between flow experience and the intention to use. Therefore, it is hypothesized that:

*H12a*: Gender moderates the relationship between flow experience and the intention to use.

*H12b*: Habituation moderates the relationship between flow experience and the intention to use.

## 3 Methods

### 3.1 Participants

Wenjuanxing^1^, an online questionnaire platform, was employed to collect data from February 4 to 21, 2025. Data collection employed a convenience sampling approach combined with snowball sampling. The questionnaire was distributed to potential participants through WeChat and QQ, two widely used social platforms in China with 1.327 billion and 558 million monthly active users (MAUs), respectively. Through two primary channels: (a) snowball sampling where initial participants were encouraged to share the survey with their peers, and (b) posts in public WeChat and QQ groups focused on online learning. Participation in the survey was voluntary and anonymous, ensuring the confidentiality and integrity of the responses. Participants were included if they: (a) were 18 years or older, (b) had used online study rooms at least once in the past 3 months, (c) were able to read Chinese, and (d) voluntarily agreed to participate. Exclusion criteria included: (a) incomplete responses, missing more than 20% of items, (b) patterned responses, (c) unrealistically short completion time (less than 60 s), and (d) duplicate submissions. A total of 408 questionnaires were completed, with 19 invalid responses excluded due to incomplete or inconsistent answers. Consequently, 389 valid responses were utilized for analysis, representing an effective response rate of 95.3%. In terms of gender, female (*N* = 272, 69.92%), male (*N* = 117, 30.08%).

The demographic analysis of the participants reveals that the predominant representation of individuals aged between 18–25 years old, accounting for 75.06% of the sample (*N* = 292), followed by the age groups of 26–35 (20.31%, *N* = 79), 36–45 (3.86%, *N* = 15), and 46–55 (0.77%, *N* = 3). There is no participant aged above 56. In terms of educational background, a significant majority of participants possess junior college or bachelor’s degrees (70.95%, *N* = 276), followed by participants with master’s degrees or higher (19.79%, *N* = 77). 3.34% of the participants have a high school degree or below (*N* = 13). These findings are consistent with the typical demographic profile of e-learning users in China, which suggest that online learning platforms mainly attract individuals with educational credentials. Participants engage with these platforms for various purposes, including exam preparations (76.86%, *N* = 299), skill improvement (64.01%, *N* = 249), personal growth (50.64%, *N* = 197), and daily learning (45.50%, *N* = 177). Additional reported motivations include the pursuit of topics of interest (31.10%, *N* = 121), language learning (16.97%, *N* = 66), and vocational training (9.25%, *N* = 36). The emphasis on exam preparation and skill improvement is consistent with the educational background distribution, which reflects a pronounced inclination toward self-learning among well-educated individuals. Overall, the demographic information and usage patterns of the collected samples correspond closely to the typical characteristics of e-learning users, supporting the appropriateness of our sample for investigating this population. High proportion of female participants (69.92%) provides sufficient statistical power for moderation analysis (male *n* = 117, female *n* = 272, exceeding the recommended 30–50 per subgroup; Cohen, 1988). We acknowledge that this gender distribution may limit generalizability to populations with different gender compositions, and we have addressed this limitation in the Discussion section. Participants’ detailed demographic information is presented in Table 1.

**Table 1:** Demographic information of the participants.

Characteristics	Frequency (*n* = 389)	Percentage (%)
Gender		
Male	117	30.08
Female	272	69.92
Age		
18–25 years old	292	75.06
26–35 years old	79	20.31
36–45 years old	15	3.86
46–55 years old	3	0.77
Over 56 years old	0	0
Educational Background		
Senior middle school or below	13	3.34
Junior college and bachelor’s degrees	276	70.95
Master’s degrees or higher	77	19.79
Others	23	5.91
Purpose of Use (Multiple Choices)		
Exam preparation	299	76.86
Skills improvement	249	64.01
Personal growth	197	50.64
Hobby learning	121	31.10
Daily learning	177	45.50
Language learning	66	16.97
Vocational training	36	9.25
Others	19	4.88

### 3.2 Instrument development

The questionnaire design of this study rigorously adhered to established academic research standards. A five-point Likert scale, ranging from 1 (strongly disagree) to 5 (strongly agree), was employed to quantify the subjective experiences of respondents. The questionnaire is structured into four sections: Technology Acceptance Model (TAM), Flow Experience (FE), Intention to use (IU), and Habituation (HAB). All items in the questionnaire were adapted from previously validated scales to ensure the scientific rigor and validity of the measurement tools. Specifically, the TAM section was used to measure perceived usefulness (PU) and perceived ease of use (PEOU), with learning convenience (LC), interactivity (INT), and the incentive and constraint mechanism (ICM) as external variables. All items in the TAM section were adapted from previous studies (Davis, 1989; Venkatesh et al., 2003; Cheung and Lee, 2011). In the FE section, adaptations from Erhel and Jamet (2019) were utilized to evaluate participants’ flow experience when using the online learning platform. Both the IU and HAB sections were adapted from Saroia and Gao (2019) and Tang et al. (2021). The IU section aimed to evaluate participants’ intention to use such platforms, and the HAB section targeted at evaluating participants’ habituation to these platforms. Moreover, the questionnaire includes a demographic section for collecting data on participants’ gender, age, educational background, and usage purposes. Prior to the formal distribution, the questionnaire was pre-tested by experts and researchers, and the wording and structure of the questionnaire were refined to ensure clarity and relevance. The final questionnaire has passed the reliability and validity analyses, confirming its overall reliability and validity.

### 3.3 Data analysis

Data were analyzed using SPSS 27.0 and AMOS 24.0. Prior to hypothesis testing, a confirmatory factor analysis (CFA) was conducted to evaluate the validity of the latent variables. In addition, Cronbach’s alpha (*α*) was calculated to assess the internal consistency of all subscales in the instrument. Subsequently, structural equation modeling (SEM) was performed to examine the relationships among the eight latent variables. SEM is particularly suited for this purpose as it accounts for measurement error and allows simultaneous estimation of multiple relationships. According to Byrne and Van De Vijver (2010), the recommended sample size for conducting CFA is 5 to 10 times the total number of items in the survey scales. In this study, there are 24 items in total, and we used 389 samples for both CFA and SEM analyses.

In the analysis process, this study employed the PROCESS macro in SPSS 27.0 specifically for moderation analysis. While SEM can also test moderation effects, PROCESS provides more detailed outputs including simple slope analysis, conditional effects at different moderator levels, and bootstrap confidence intervals for the interaction terms. The number of bootstrap resamples was set to 5,000 to ensure the robustness and reliability of the results and to minimize the impact of sampling error on the analysis outcomes. The confidence interval was set at 95% to assess the significance of the moderation effects. PROCESS Model 1 was used to systematically examine the moderating roles of gender and habituation. Through these analytical procedures and parameter settings, the study can comprehensively and systematically evaluate the moderating effects of gender and habituation on the relationships between perceived ease of use, perceived usefulness, flow experience, and intention to use.

## 4 Results

In this study, the factor loadings of all 24 items ranged from 0.700 to 0.905. Cronbach’s alpha coefficients for the subscales of LC, INT, ICM, PU, PEOU, FE, HAB, and IU were 0.717, 0.789, 0.700, 0.765, 0.778, 0.709, 0.813, and 0.905, respectively. These values indicate good internal consistency across the subscales. The factor loadings for each item and the Cronbach’s alpha values for each subscale are presented in Table 2.

**Table 2:** Results of construct validity and reliability analysis.

Latent variable	Measurement variable	Mean	Std. dev	Factor loadings	Alpha	CR	AVE
LC	LC1	4.414	0.591	0.688	0.717	0.719	0.461
	LC2			0.631			
	LC2			0.683			
INT	INT1	4.116	0.759	0.737	0.789	0.79	0.557
	INT2			0.732			
	INT3			0.729			
ICM	ICM1	4.205	0.672	0.641	0.700	0.701	0.439
	ICM2			0.658			
	ICM3			0.668			
PU	PU1	4.237	0.656	0.717	0.765	0.766	0.522
	PU2			0.713			
	PU3			0.727			
PEOU	PEOU1	4.239	0.640	0.716	0.778	0.779	0.54
	PEOU2			0.634			
	PEOU3			0.630			
FE	FE1	4.144	0.736	0.726	0.709	0.709	0.449
	FE2			0.716			
	FE3			0.712			
IU	IU1	4.302	0.740	0.719	0.813	0.814	0.593
	IU2			0.781			
	IU3			0.795			
HAB	HAB	3.111	1.242	0.874	0.905	0.905	0.76
	HAB			0.850			
	HAB			0.895			

### 4.1 Assessment of the model fit

As demonstrated in Table 3, the fit indices of the measurement and structural models confirmed the validity of the employed constructs. Although the model fit indices (AGFI = 0.814, TLI = 0.826, CFI = 0.853) were slightly below the recommended thresholds, they are considered acceptable in exploratory studies (Hu and Bentler, 1999). While the RMR (0.160) exceeds the conventional 0.05 cutoff, this metric’s sensitivity to parameter scales makes it less reliable than RMSEA in complex models (Bentler, 2006). We acknowledge that these values do not reach the ideal benchmarks, which may be attributed to the complexity of our integrated model combining TAM and Flow Theory, an understudied theoretical integration requiring cross-paradigm adjustments. Our RMSEA value (0.089) falls within the “mediocre fit” range (0.08–0.10), as defined by MacCallum and Hong (1997), which remains acceptable when: (a) Sample size smaller than 500 (*N* = 389 in this study); (b) Model includes over 20 observed variables (24 items here). Future research should consider alternative model specifications or additional constructs to improve model fit. As emphasized by Barrett (2007), model evaluation should prioritize theoretical coherence over mechanical cutoff adherence (Maydeu-Olivares, 2017; McIntosh, 2017).

**Table 3:** The goodness of fit indices for the measurement model and the research model.

Model	χ^2^/df	AGFI	TLI	CFI	RMR	RMSEA
Measurement model	1.659	0.905	0.960	0.967	0.032	0.041
Research model	4.078	0.814	0.826	0.853	0.160	0.089
Recommended value references	<5.0	>0.90	>0.90	>0.90	<0.05	<0.08

### 4.2 Hypotheses testing

The significant correlations among LC, INT, ICM, PU, PEOU, FE, and IU indicate that these variables are highly interconnected. To examine the structural relationships among these variables, Structural Equation Modeling (SEM) was conducted to test the significance of each hypothesized path. The analysis calculated non-standardized coefficients (B), standardized coefficients (*β*), standard errors (SE), *t*-value, and R2 (explanatory power of independent variables) were calculated to test the hypotheses. The results indicated that ten out of twelve hypotheses were supported (Table 4; Figure 2).

**Table 4:** The results of hypothesis testing.

Hypotheses	Hypothesized path	B	β	SE	CR	Supported
H1	Perceived Ease of Use- > Perceived Usefulness	−0.101	−0.101	0.139	−0.728	Not supported
H2	Perceived ease of use-- > Intention to Use	−0.022	−0.017	0.231	−0.095	Not supported
H3	Perceived usefulness-- > Intention to Use	0.329	0.262	0.149	2.209	Supported
H4	Learning Convenience--- > Perceived Ease of Use	0.447	0.504	0.067	6.628***	Supported
H5	Learning Convenience--- > Perceived Usefulness	0.489	0.461	0.091	4.469***	Supported
H6	Interactivity--- > Perceived Ease of Use	0.240	0.357	0.044	5.430***	Supported
H7	Interactivity--- > Perceived usefulness	0.461	0.622	0.059	7.130***	Supported
H8	Interactivity--- > Flow Experience	0.599	0.747	0.057	10.478***	Supported
H9	Incentive and Constraint Mechanism--- > Perceived Ease of Use	0.437	0.489	0.071	6.180***	Supported
H10	Incentive and Constraint Mechanism--- > Perceived Usefulness	0.615	0.691	0.103	5.984***	Supported
H11	Incentive and Constraint Mechanism-- > Flow Experience	0.701	0.659	0.085	8.257***	Supported
H12	Flow Experience-- > Intention to Use	0.416	0.395	0.147	2.830	Supported

^***^p < 0.001; **p < 0.01; *p < 0.05.

**Figure 2:**
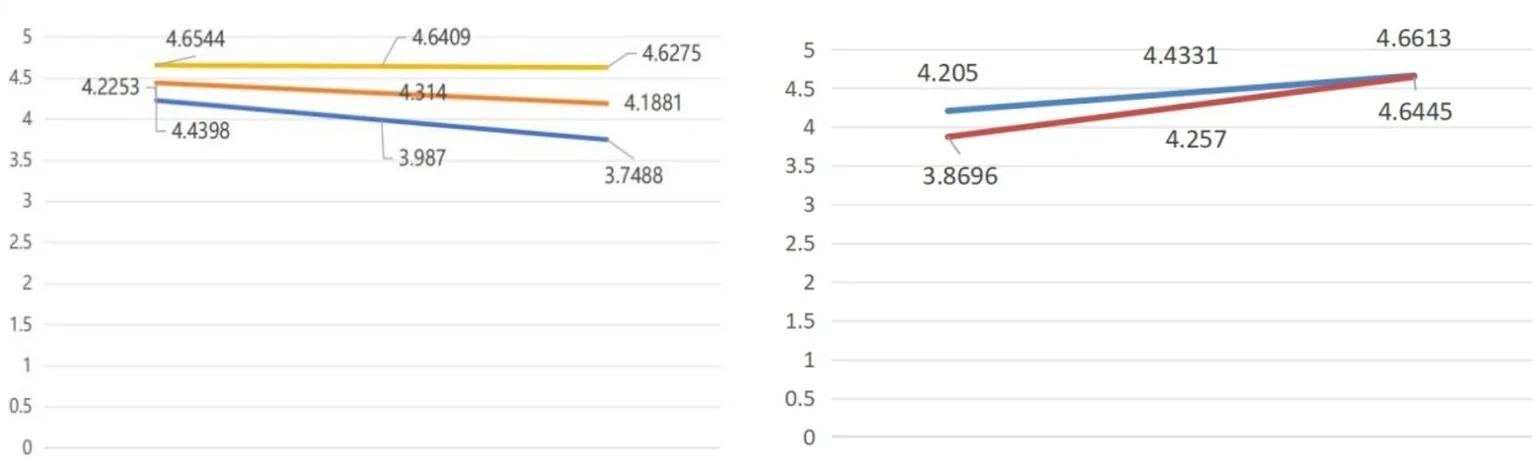
The simple slope plot of the moderation analysis of GEN and HAB on the relationship between PEOU and IU.

Learning Convenience was found to have significant positive direct effects on Perceived Ease of Use (*β* = 0.504, *p* < 0.001), and Perceived usefulness (*β* = 0.461, *p* < 0.001), supporting Hypothesis 4 and Hypothesis 5. Interactivity was found to have significant positive direct effects on Perceived Ease of Use (*β* = 0.357, *p* < 0.001), Perceived Usefulness (*β* = 0.622, *p* < 0.001), and Flow Experience (*β* = 0.747, *p* < 0.001), supporting Hypothesis 6, Hypothesis 7 and Hypothesis 8. The Incentive and Constraint Mechanism was found to have significant positive direct effects on Perceived Ease of Use (*β* = 0.489, *p* < 0.001), Perceived Usefulness (*β* = 0.691, *p* < 0.001), and Flow Experience (*β* = 0.659, *p* < 0.001), supporting Hypothesis 9, Hypothesis 10 and Hypothesis 11. Perceived Ease of Use was found to have significant positive direct effects on Intention to Use (*β* = 0.262, *p* < 0.001), supporting Hypothesis 2. Flow Experience was found to have significant positive direct effects on Intention to Use (*β* = 0.395, *p* < 0.001), supporting Hypothesis 12.

However, Perceived Ease of Use was not found to have significant direct effects on Perceived Usefulness (*β* = −0.101, *p* = −0.728), rejecting Hypothesis 1. Also, Perceived Usefulness was not found to have significant direct effects on Intention to Use (*β* = −0.017, *p* = −0.095), rejecting Hypothesis 3.

### 4.3 Results of moderating effects

Table 5 presents the analysis of the moderating effects of gender (GEN) and habituation (HAB) on PEOU and IU. As shown in the table, the interaction term between PEOU and HAB (PEOU*HAB) has a significant influence on IU (*β* = −0.115, *p* = 0.013), indicating the moderation of HAB on the relationship between PEOU and IU. The moderating effect is negative, which suggests that as the level of HAB increases, the positive influence of PEOU on IU diminishes. In contrast, though the interaction term between PEOU and GEN (PEOU*GEN) shows a negative trend (*β* = −0.092), the effect is only marginally significant (*p* = 0.061), which did not reach the conventional significance threshold (*p* < 0.05). In summary, PEOU has a robust positive predictive influence on IU and HAB significantly and negatively moderates this relationship. However, the moderating effect of GEN is relatively weak.

**Table 5:** Analysis of the moderating effects of GEN and HAB on the relationships between PEOU and IU.

	Unstandardized coefficient (B)	Standard error	Standardized coefficient beta	*t*	Significance
(Constant)	2.026	0.222		9.117	0
PEOU	0.59	0.056	0.51	10.443	0
PEOU*GEN	−0.031	0.017	−0.092	−1.88	0.061
PEOU*HAB	−0.015	0.006	−0.115	−2.504	0.013

Dependent Variable: IU.

Simple slope analysis was conducted to examine the moderating effect of habituation on the relationship between perceived ease of use and intention to use. For users with low habituation (−1 SD), the effect of PEOU on IU was *β* = 0.605 (*p* < 0.001). For users with high habituation (+1 SD), this effect was reduced to *β* = 0.575 (*p* < 0.001). This indicates that as habituation increases, the positive influence of perceived ease of use on intention to use diminishes, supporting H4a.

Table 6 investigates the moderating effects of GEN and HAB on the relationship between PU and IU. The results of regression analysis demonstrate that GEN has a significant negative moderating effect (*β* = −0.108, *p* = 0.026), which suggests that compared with female users, the positive influence of PU on IU is diminished among male users. The moderating effect of HAB is also negative (*β* = −0.087, *t* = −1.948) but it only reaches marginal significance (*p* = 0.052), which indicates that habituation may weaken PU’s positive influence on IU (see Figure 3).

**Table 6:** Analysis of the moderating effects of GEN and HAB on the relationships between PU and IU.

	Unstandardized coefficient (B)	Standard error	Standardized coefficient beta	*t*	Significance
(Constant)	1.89	0.211		8.979	0
PEOU	0.631	0.054	0.559	11.58	0
PEOU*GEN	−0.036	0.016	−0.108	−2.232	0.026
PEOU*HAB	−0.011	0.006	−0.087	−1.948	0.052

Dependent Variable: IU.

**Figure 3:**
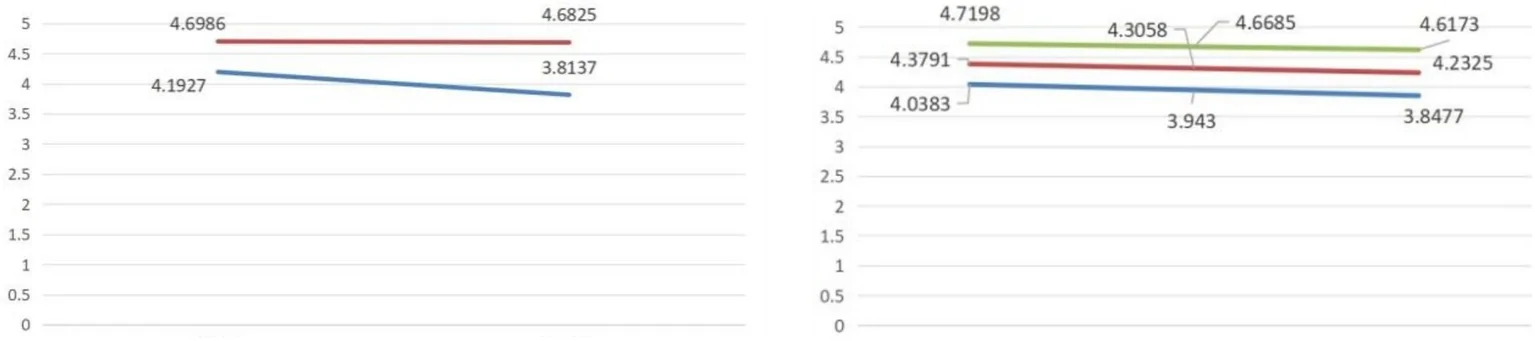
The moderating effects of GEN and HAB on the relationship between PEOU and IU.

Table 7 elucidates that the interaction term between GEN and FE (FE*GEN) exhibits a negative trend (*β* = −0.08), but it does not reach statistical significance (*p* = 0.105). Similarly, the interaction term between HAB and FE (FE*HAB) also shows a negative but non-significant moderating effect (*β* = −0.06, *p* = 0.179). These findings suggest that, within the context of this study, the moderating effects of gender and habituation on the relationship between flow experience and intention to use may be relatively weak (see Figure 4).

**Table 7:** Analysis of the moderating effects of GEN and HAB on the relationships between FE and IU.

	Unstandardized coefficient (B)	Standard Error	Standardized coefficient beta	*t*	Significance
(Constant)	2.16	0.184		11.734	0
PEOU	0.562	0.049	0.559	11.358	0
FE*GEN	−0.027	0.016	−0.08	−1.625	0.105
FE*HAB	−0.008	0.006	−0.06	−1.348	0.179

Dependent variable: IU.

**Figure 4:**
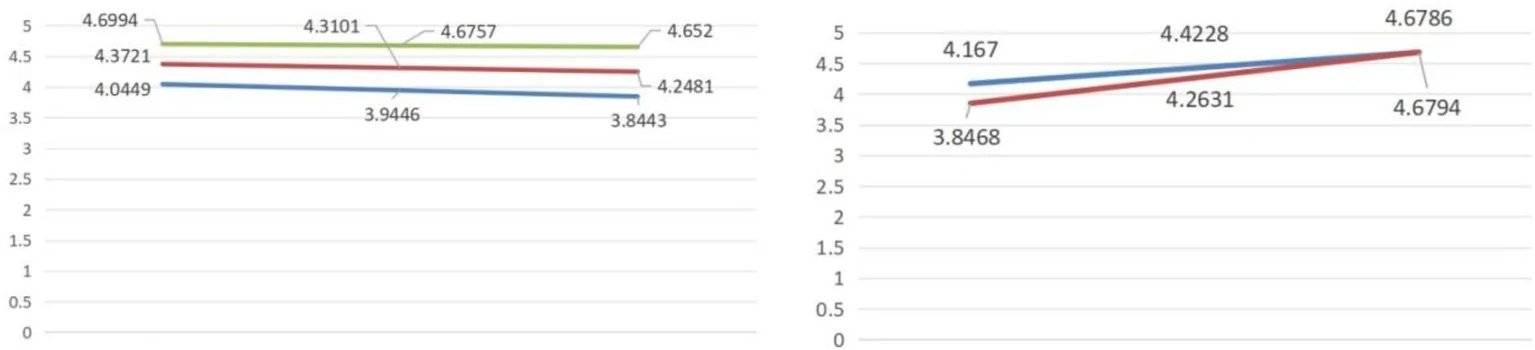
The moderating effects of GEN and HAB on the relationship between FE and IU.

## 5 Discussion

### 5.1 Integration of TAM and the flow theory

This study substantiates the combined influences of rational cognition and emotional engagement on the behavioral intention to use online study rooms, aligning with the opinions of Hsu et al. (2012), which emphasizes that user behavior is driven by both instrumental benefits and emotional rewards. Notably, our research results display that flow experience, as an emotional participation mechanism, mediates the relationships between perceived usefulness, perceived ease of use, and intention to use. This finding broadens the applicability of TAM and demonstrates that while choosing online study rooms, users weigh both the instrumental benefits offered by such platforms and the emotional rewards experienced through flow. It is worth noting that the direct influence of perceived usefulness on intention to use is not significant, which diverges from traditional TAM hypotheses and warrants critical theoretical interpretation. We propose three complementary explanations for this unexpected result.

First, drawing on Self-Determination Theory (Ryan and Deci, 2000), in emotion-driven learning contexts such as online study rooms, users’ needs for autonomy, competence, and relatedness may outweigh the instrumental benefits. Users may prioritize the sense of belonging derived from social interaction and the intrinsic enjoyment of immersive learning over utilitarian outcomes. When emotional needs are primary, the instrumental usefulness of the platform becomes secondary in predicting continued use.

Second, the hedonic versus utilitarian value framework offers additional insight. Online study rooms serve dual purposes: they are both functional tools for learning efficiency (utilitarian value) and experiential spaces for social connection and enjoyment (hedonic value). Our results suggest that in this particular context, hedonic value captured through flow experience may be the dominant driver of behavioral intention, while utilitarian value (perceived usefulness) plays a subordinate role.

Third, the demographic composition of our sample (predominantly young, well-educated users) may contribute to this finding. Contemporary users, especially the younger generation, have become highly adept at navigating digital platforms. For these users, perceived usefulness may be a necessary but not sufficient condition for adoption. Once basic usefulness is established, emotional and experiential factors become the differentiators that drive continued intention to use.

### 5.2 Extending TAM with external variables

This research unveils the significant positive influences of external variables (i.e., learning convenience, interactivity, incentive and constraint mechanism) on perceived usefulness and perceived ease of use, which coincides with the research results of Lisana (2023) and Qashou (2021) and suggests that these external variables can significantly improve user acceptance of and intention to use online learning platforms. Different from established TAM studies, the direct influence of perceived usefulness on intention to use is not significant in our research, which contrasts with previous emphasis on its central role in prior research (Davis, 1989). Through our analysis, it is posited that this shift may stem from the widespread adoption of technology and users’growing adaptability. With the popularization of Internet technologies, contemporary users, especially the young generation, have become adept at navigating complex multitasking interfaces, which may render them less sensitive to perceived ease of use. Moreover, the objectives of using online study rooms among users exhibit considerable diversification. Apart from improving learning efficiency, some users emphasize the emotional support and the sense of belonging derived from interaction and socializing. This diversification can lead to a corresponding variation, which may influence the relative importance of perceived usefulness across different user segments. According to the Self-Determination Theory, in emotionally driven learning contexts, user demands for autonomy and a sense of control may outweigh the pursuit of instrumental benefits. Therefore, these users are more inclined to favor platforms that can satisfy their emotional needs instead of solely seeking those that provide instrumental benefits.

### 5.3 Analysis of the moderating effects of gender and habituation

This research further discusses the moderating effects of GEN and HAB on the relationships between PEOU, PU, FE, and IU. The discovery of the moderating effects enhances our understanding of the mechanisms underlying users’ behavioral intentions and uncovers the significant role of individual differences in decision-making processes.

#### 5.3.1 The moderating effect of gender (GEN)

Our research results exhibit that gender has a significant negative moderating effect on the relationship between PU and UI (*β* = −0.108, *p* = 0.026). Specifically, the positive influence of PU on UI is weaker among male users compared to that among female users. This finding indicates that gender differences can influence the behavioral intention to use online study rooms. Previous literature has also revealed that male users tend to prioritize instrumental benefits and functional values of technologies while female users place greater emphasis on emotional experiences and social interactions (Eagly and Wood, 1999). Consequently, in the context of online study rooms, female users’ intention to use may be enhanced more through flow experiences and emotional rewards, whereas male users may prioritize instrumental benefits. This finding offers new evidence for the influence of gender differences on user behavior and suggests that product designers and marketers should consider gender-specific strategies to better cater to diverse user needs.

#### 5.3.2 The moderating effect of habituation (HAB)

Habituation has a significant negative moderating effect on the relationship between PEOU and UI (*β* = −0.115，*p* = 0.013), which indicates that as users’ level of habituation level increases, PEOU’s positive influence on UI diminishes. This result demonstrates that habituation can weaken users’ sensitivity to PEOU. According to (Verplanken and Orbell, 2022), habituation is characterized as an automated behavioral mode. Once developed, decision-making processes could be more driven by habituation rather than through rational evaluation. Within the context of online study rooms, users may develop usage habits over time and their attention to PEOU can be reduced. This finding highlights the critical role of habituation in decision-making processes and suggests that product designers can improve user satisfaction and intention to use by designing functions that suit users’ habituated behaviors (see Figure 5).

**Figure 5:**
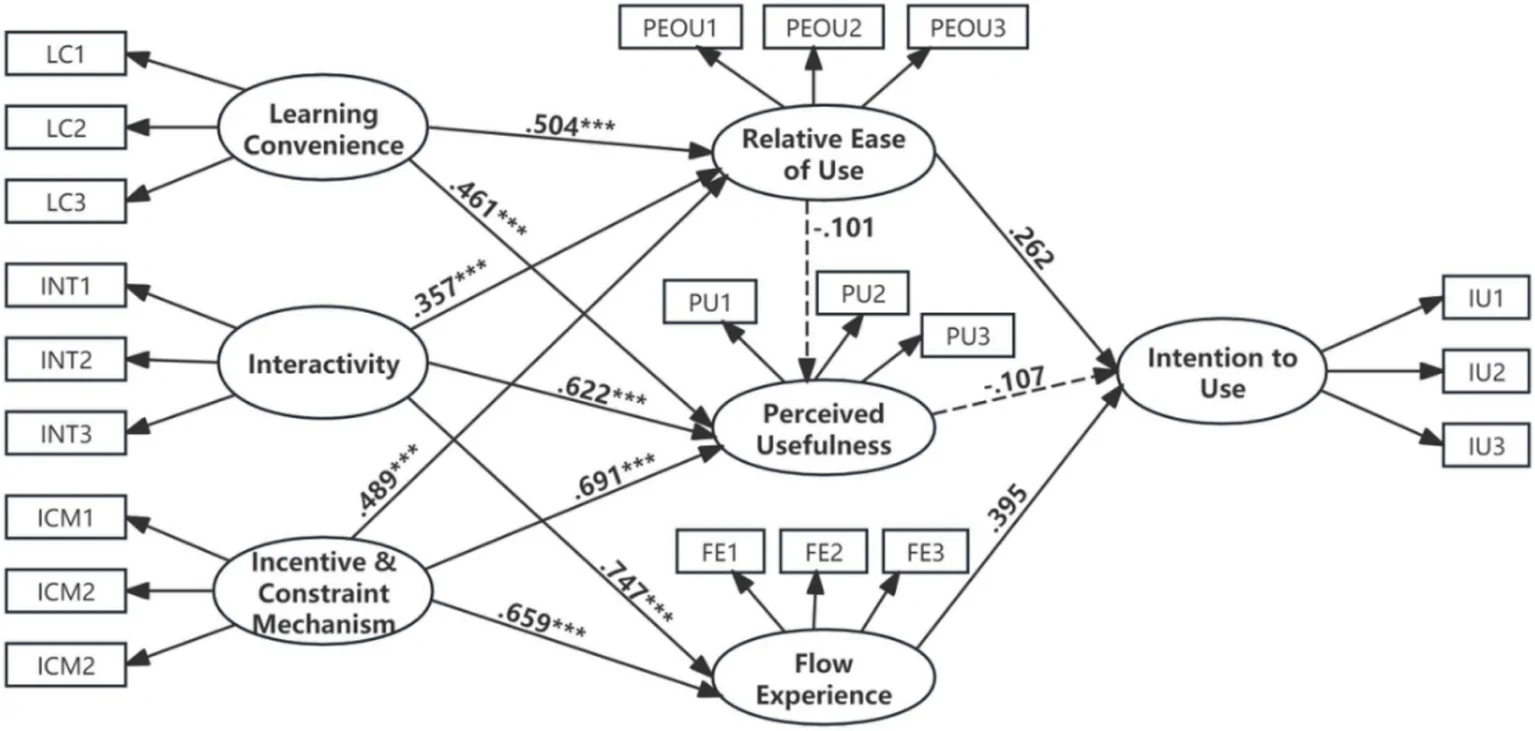
Hypotheses test results.

## 6 Contribution and Implication

### 6.1 Theoretical contribution

#### 6.1.1 Integration of TAM and flow theory

This research establishes a cross-theory framework by integrating TAM and the Flow Theory to elucidate the behavioral intention to use online study rooms. This framework integrates emotional experiences (such as flow experience) with rational cognitive processes, which addresses gaps in extant literature. Traditional TAM studies have predominantly focused on the cognitive dimension of perceived usefulness and perceived ease of use. By introducing the flow experience as an emotional driver, this study unveils a more comprehensive perspective for understanding user behavior in online learning environments. This innovation offers a novel analytical tool for online learning scenarios that involve emotional and cognitive interactions.

#### 6.1.2 Expand the external variable of TAM

This research validates the moderating effects of external variables (i.e., learning convenience, interactivity, and the incentive and constraint mechanism) in TAM, which expands the applicability of TAM. By introducing the socializing features and the behavioral supervision mechanism, this research reveals how these external variables influence user behaviors through dual pathways of emotion and cognition. This finding aligns with the research results of (Cheng and Jiang, 2020), which highlights the enhancement of concentration via real-time interactions. Furthermore, this study offers new theoretical insights for the application of TAM in the domain of online education.

#### 6.1.3 Revelation of the moderating effects of gender and habituation

This research broadens the theoretical scope of TAM by investigating how gender and habitual behaviors moderate the technology acceptance trajectory of engaging with online learning platforms. First, the incorporation of gender and habituation offers a more nuanced framework for understanding the mechanisms underlying behavioral intention formation and unveils the essential role of individual differences in decision-making processes. Second, the identification of these moderators provides new theoretical support for the application of TAM in online learning scenarios. It is highlighted that user behaviors are not only influenced by instrumental benefits and emotional rewards but also influenced by individual differences, such as gender and habituation. By revealing these moderating effects, this research not only enriches the theoretical connotation of TAM but also offers a new theoretical lens and analytical framework for studying user behaviors in the context of online learning environments.

### 6.2 Practical implications

#### 6.2.1 Optimization of online study room design

In the design of online study rooms, platform operators are encouraged to optimize functionalities in response to gender differences. For female users, more interest-based learning groups can be established for them to socialize and enhance emotional exchanges and mutual support; while for male users, the emphasis should be placed on enhancing learning efficiency through the provision of tools such as intelligent learning plan generators and learning data analysis. For the habituation part, it is essential to leverage users’ historical data to provide personalized study paths. In this way, users can better adapt to the platforms and improve their learning outcomes. At the same time, the interactive features should be continuously optimized–such as improving bullet comment rules, real-time Q&A mechanisms, and users’ flow experience in learning.

#### 6.2.2 Improvement of promotional strategies to boost the intention to use

Our research results offer valuable insights for online learning platforms aiming to develop more effective promotional strategies. For users who prioritize emotional experiences, platforms should highlight the interactive features and social atmosphere; for users who seek to improve learning efficiency, a focus on practical functionalities that facilitate efficient learning should be emphasized; for novice users, such platforms should shed light on the ease of use, basic functionalities, and advantages; while for experienced users, emphasis should be placed on the advanced features and personalized services. Moreover, online learning activities, such as check-in challenges and group competitions, can be held to attract users of different types and enhance user stickiness and frequency of use. Furthermore, differentiated features and services tailored to the distinct needs of users of different genders should be provided. Meanwhile, it is also recommended to encourage users to cultivate positive learning habits to improve their reliance on and loyalty to the platform.

#### 6.2.3 Offering analysis results for online education

The integrated model in our study serves as a systematic instrument for user behavior analysis in online education. By quantifying the influences of learning convenience, interactivity, and the incentive and constraint mechanism on user behavior, online learning platforms can identify user demands more accurately, optimize their function designs accordingly, and formulate corresponding data-driven operational strategies. Diversified online learning scenarios applicable to multi-task interfaces and social interaction in this research offer scientific evidence for the application of educational technologies.

## 7 Limitations and further research

This research integrates TAM and the Flow Theory to examine the driving forces of users’ behavioral intention to use online study rooms. Despite its contributions, there exist some limitations. First, the research data are predominantly from China, which may impose cultural limitations. Future research can further explore the behavioral differences among users of online study rooms against different cultural backgrounds, thereby offering more robust theoretical support for the internationalization of such platforms. Second, this study relies on cross-sectional data and views the flow experience as a static outcome, which limits the ability to capture the dynamic change of flow thresholds. To this end, future research can conduct multi-modal data analyses, such as integrating eye-tracking and log data, to conceptualize flow experience as a dynamic variable to understand user behavior and psychological changes.

## 8 Funding sources

This research did not receive any specific grant from funding agencies in the public, commercial, or not-for-profit sectors.

Questionnaire items and their source of adoption.

**Table:** 

Variables	Items	Measurement items	Adapted from
Learning Convenience (LC)	LC1	Online study rooms allow me to start learning anytime anywhere.	Hsu et al. (2012)
	LC2	I can flexibly arrange my learning plan based on my schedule.	
	LC3	Online study rooms support switching between multiple devices (such as smartphones, tablets, and computers), which is convenient to use.	
Interactivity (INT)	INT1	In online study rooms, I can interact with other learners via bullet comments and comments, which can enrich my learning experience.	Venkatesh et al. (2003)
	INT2	The study group feature in online study rooms gives me a sense of peer support.	
	INT3	I am influenced by the host of the online study room, which makes me more willing to participate in study sessions.	
Incentive and Constraint Mechanism (ICM)	ICM1	The progress tracking feature in online study rooms helps me better understand my learning achievements.	Cheung and Lee (2011)
	ICM2	The achievement system of online study rooms (such as badges and leaderboards) motivates me to keep studying.	
	ICM3	Online study rooms foster a sense of achievement through interactions via bullet comments and comments, motivating me to continue learning.	
Flow Experience (FE)	FE1	I can completely concentrate on my learning tasks and lose track of time when I am using online study rooms.	Séverine Erhel & Eric Jamet (2019)
	FE2	The virtual environment of online study rooms (such as background music and study scenes) gives me a sense of immersion and helps me focus better on my learning tasks.	
	FE3	The bullet comment interactions and check-in feature of online study rooms enhance my sense of engagement and immersion, making me more willing to learn.	
Perceived Usefulness (PU)	PU1	The functional designs of online study rooms help me better manage my time.	Davis, (1989)
	PU2	My learning efficiency has improved after using online study rooms.	
	PU3	Online study rooms are very helpful for realizing my long-term study goals.	
Perceived Ease of Use (PEOU)	PEOU1	The operations of online study rooms are very easy.	Davis, (1989)
	PEOU2	I can quickly master the functions of online study rooms.	
	PEOU3	It does not take too much time to learn how to use online study rooms.	
Habituation (HAB)	HAB1	For me, choosing to study offline is an automatic behavior.	Tang et al. (2021)
	HAB2	Studying offline (e.g., using a fixed seat and taking handwritten notes) feels very natural to me and barely requires deliberate planning.	
	HAB3	When I need to focus on studying, I would prefer to study offline.	
Intention to Use (IU)	IU11	Recently, I had the intention to use online study rooms.	Saroia and Gao, (2019)
	IU2	If possible, I tend to use online study rooms to study.	
	IU3	If possible, I plan to shift from studying offline to studying in online study rooms.	

## Data Availability

The datasets presented in this study can be found in online repositories. The names of the repository/repositories and accession number(s) can be found at: the datasets generated and/or analysed during the current study are available in the Mendeley Data repository, https://doi.org/10.17632/gcpptfds6y.1.

## Footnotes

1
https://www.wjx.cn/
